# Introducing the “Corona Curtain”: an innovative technique to prevent airborne COVID-19 exposure during emergent intubations

**DOI:** 10.1186/s13037-020-00247-5

**Published:** 2020-05-13

**Authors:** Eric Hill, Christopher Crockett, Ryan W. Circh, Frank Lansville, Philip F. Stahel

**Affiliations:** 1 The Medical Center of Aurora, 1501 South Potomac St, Aurora, CO 80012 USA; 2 Covenant Health, Methodist Medical Center of Oak Ridge, 990 Oak Ridge Turnpike, Oak Ridge, TN 37831 USA; 3 Department of Specialty Medicine, Rocky Vista University, College of Osteopathic Medicine, Parker, CO 80134 USA

**Keywords:** COVID-19, SARS-CoV-2, Coronavirus, Intubation, Viral exposure

## Abstract

**Background:**

The coronavirus disease 2019 (COVID-19) pandemic places healthcare workers at risk of exposure to the severe acute respiratory syndrome coronavirus-2 (SARS-CoV-2). Emergency department (ED) staff are particularly vulnerable when managing patients with acute respiratory distress due to the aerosolization of the virus during endotracheal intubation. A simple and innovative intubation tent was designed with the intent of decreasing the risk of accidental viral transmission from emergent intubations during the COVID-19 pandemic.

**Presentation of technique:**

The materials and assembly process of the novel “Corona Curtain” are described in technical detail, with the intent of allowing other providers to template the concept at their respective facilities.

**Results:**

A total of 36 intubation tents were mounted in the ED at the Medical Center of Aurora, Colorado, on April 7, 2020, and thereafter consistently used for all intubations during the ongoing COVID-19 outbreak. The cost of raw materials and labor for the initial assembly averaged US $ 8.00 per construct. The price of the single-use plastic cover is variable depending on the vendor source.

**Conclusion:**

The new “Corona Curtain” was designed to improve the safety of ED staff when performing urgent/emergent intubations during the current COVID-19 pandemic. The concept can easily be adopted to other patient care areas, including perioperative and intensive care units. Future validation studies are needed to determine the safety and efficacy of the intubation tents by quantifying the pre−/post-intubation exposure through “point-of-care” SARS-CoV-2 testing once these resources are more widely available.

## Background

The current pandemic of the novel coronavirus disease 2019 (COVID-19) imposes a significant risk of viral transmission to healthcare workers who take care of infected patients, with high reported mortality rates [1–4]. Between February 12 and April 9, 2020, nearly 10,000 COVID-19 cases of infected healthcare personnel in the United States were reported to the Center for Disease Control and Prevention (CDC), with a median age of 42 years [5]. While most infected healthcare professionals are not hospitalized, severe adverse outcomes, including death, have been reported in all age groups [5]. Our current understanding of the mechanisms of viral transmission of the severe acute respiratory syndrome coronavirus-2 (SARS-CoV-2) in the healthcare setting remains limited [6]. The predominant assumption is that SARS-CoV-2 is transmitted by droplets or contact from respiratory secretions, however, airborne transmission may occur under certain high-risk circumstances [7–9]. The general recommendations regarding the use of personal protective equipment (PPE) for contact with patients who are either confirmed or suspected of SARS-CoV-2 infection include fluid-resistant gowns, masks, gloves, and goggles [10–12]. Aerosolizing procedures require wearing full face shields and fit-tested N95 respirators, or alternatively powered air-purifying respirators (PAPRs) [13]. The high-risk aerosol-generating conditions include noninvasive positive pressure ventilation (NPPV) and high-flow nasal cannula (HFNC) oxygenation, nebulizer treatment, sputum induction, bronchoscopy, and endotracheal intubation or extubation [11–13]. In these specific instances, patients should be isolated in a negative-airflow isolation room, if available, or alternatively be placed in a single isolation room with closed doors [11–13]. The use of PAPRs provides intuitive benefits over N95 masks combined with face shields, including the comfort of wearing PAPRs during prolonged resuscitations and the additional safety of circumferential coverage with increased protection from accidental contact exposure [13]. In addition, the so-called hazardous materials (“hazmat”) suits, technically termed “encapsulated impermeable chemical protective suits”, provide a safe alternative option for emergent intubations and resuscitations in the ED [14]. Practical recommendations and consensus guidelines for protecting staff and providers from aerosol exposure during endotracheal intubations of presumed COVID-positive patients have been presented in multiple recent publications [15–18]. The recommended safety precautions include the standardized use of video-assisted laryngoscopy for endotracheal intubations to attenuate the risk of aerosol exposure by increasing the distance between provider and patient during the procedure [12, 13, 18, 19]. An additional prevalent strategy to decrease the risk of accidental viral exposure during in−/extubation is to limit surgical cases during the COVID-19 pandemic by risk-stratification to essential indications exclusively, and to take all necessary precautions in the perioperative management of urgent and emergent cases [20–22].

In light of the widespread prevalence of COVID-19 in the community (at the time of drafting of this article), every ED patient requiring urgent or emergent intubation is considered to be potentially SARS-CoV-2 positive and managed according to the published precautions [22–25]. At our institution, emergent intubations are preferably performed in negative airflow rooms with the intubating physician wearing a “hazmat” suit and the assisting ED nurse and respiratory therapist wearing full PPE, goggles, face shields, and N95 masks. We recently introduced an additional safety measure by incorporating novel “intubation tents” to all ED bays at our hospital, with the intent of further decreasing exposure to aerosol generation and COVID-19 transmission during emergent intubations. The present article provides an overview on the design and assembly technique of the “Corona Curtain” to allow other providers and facilities to template and validate the application of this innovative, simple and cheap safety strategy during the current COVID-19 pandemic.

## Presentation of technique

### Materials

The “Corona Curtain” is built with common, low-price plumbing materials available from community hardware stores. These include the following specific items:

- Cross-linked polyethylene (PEX) tubes of ¾ inch (1.9 cm) diameter, cut to a length of 6 ft (1.8 m) and 10 ft (3 m), respectively. Two tubes of either length are needed for the assembly of one tent.
- Copper pressure materials (× 2 for one tent):

- Two air chambers of ½ in. (1.3 cm) diameter and 8 in. length (20 cm).
- One 45° curved coupler of ½ in. (1.3 cm) diameter.
- One T-type coupler of ½ in. (1.3 cm) diameter.
- One diameter-reducing coupler of ½ in. (1.3 cm) to 3/8 in. (1 cm) diameters.
- One copper coil tube of 3/8 in. (1 cm) diameter and 3 in. length (7.6 cm).

c. Plastic drape of size 10 ft × 12 ft (3 m × 3.7 m), to cut off a roll at the appropriate length (for example, use D250 bagging film with temperature rating of 200 °F/93 °C).
d. One extra-large binder clip.

### Assembly

**Fig. 1:**
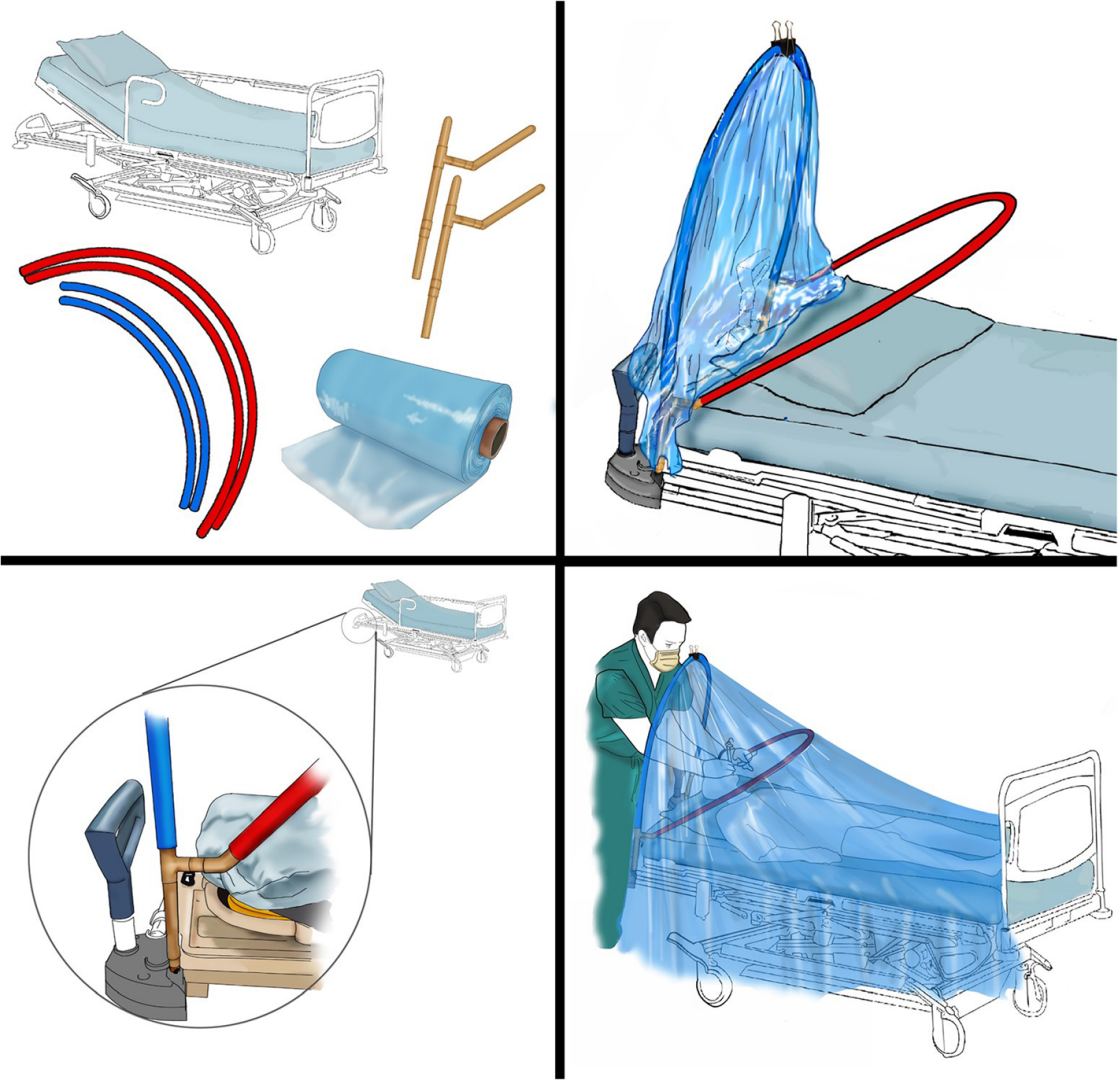
Schematic depiction of the “Corona Curtain” principle. See text for details.© Phil Stahel 2020

**Fig. 2:**
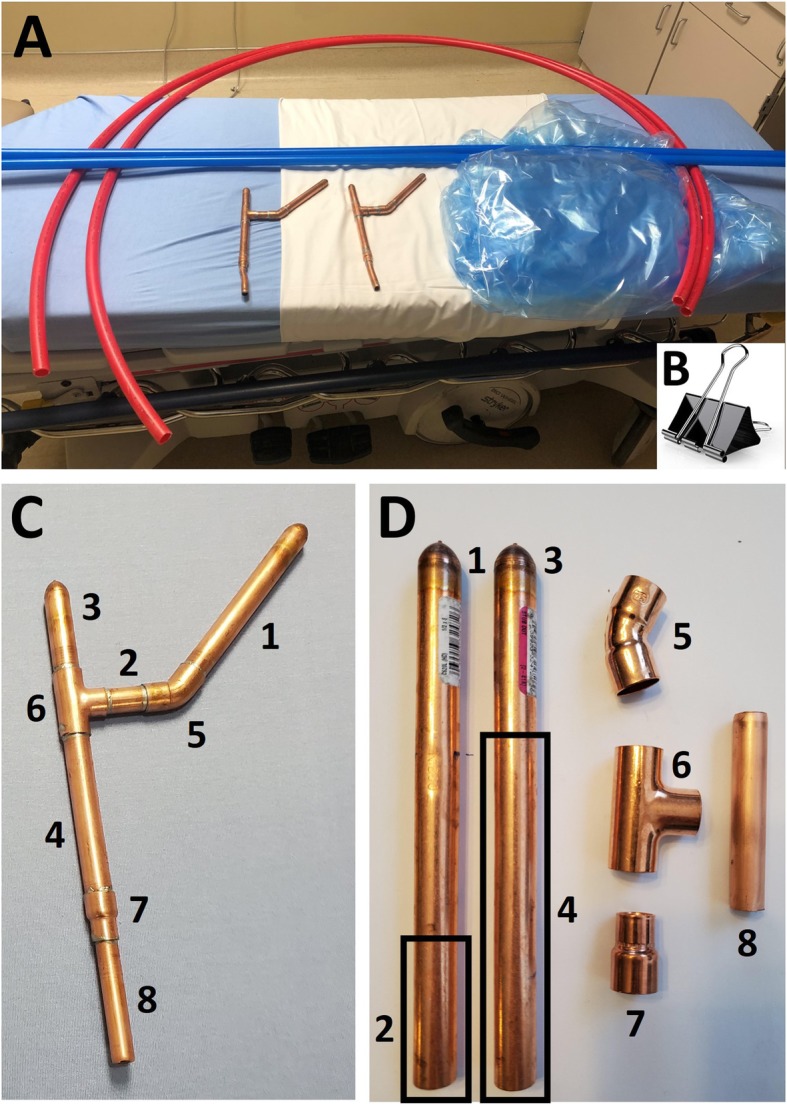
Materials required for assembly of the intubation tent (**a,b**). The copper posts (**c**) are welded from the individual copper pressure air chamber pieces (**d**). Legend: 1 – Copper pressure air chamber of 8 in. length (20 cm). 2 – Fragment of 2 in. length (5 cm) cut off the open end of air chamber 1. 3 – Copper pressure air chamber of 8 in. length (20 cm). 4 – Fragment 4 ¾ in. (12 cm) cut off the open end of air chamber 3. 5 – Curved 45° coupler of ½ in. (1.3 cm) diameter. 6 – T-type coupler of ½ in. (1.3 cm) diameter. 7 – Reducing coupler of ½ in. (1.3 cm) to 3/8 in. (1 cm) diameters. 8 – Coil tube of 3/8 in. (1 cm) diameter, 3 in. length (7.6 cm)

The schematic drawing in Fig. 1 depicts the assembly steps for the “Corona Curtain”. The specific underlying materials are shown in Fig. 2. The PEX tubes are cut at a length of 6 ft (blue tubes) and 10 ft (red tubes). Distinct tube colors were selected to allow easy differentiation of the two sizes in daily practice. The two copper pressure air chambers of 8 in. length (20 cm) are cut at the following distinct lengths:

- Cut 2 in. (5 cm) off the open end (#2 in Fig. 2, panel d), and use the residual part for the 45° riser (#2 in Fig. 2, panel c).
- Cut 4 ¾ inches (12 cm) off the open end (#4 in Fig. 2, panel d) and use the residual part for the vertical riser (#4 in Fig. 2, panel c).

**Fig. 3:**
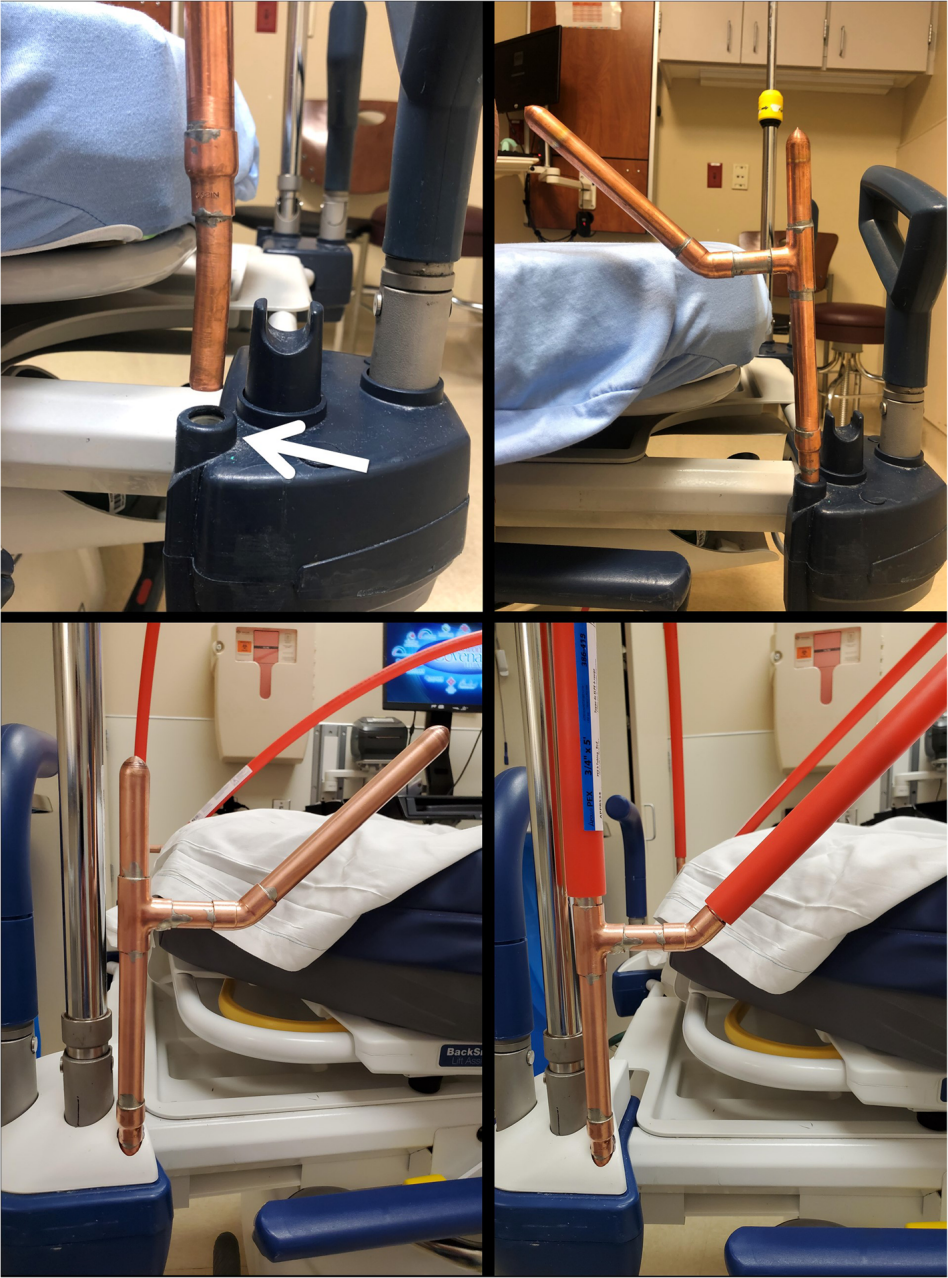
Mounting of the copper posts and PEX tubes to commonly used patient beds in the emergency department. The posts are inserted to the bed through the 3/8 in. (1 cm) diameter connecting coil tube (arrow in upper left panel). The PEX tubes are inserted to the receiving ends of the copper posts

**Fig. 4:**
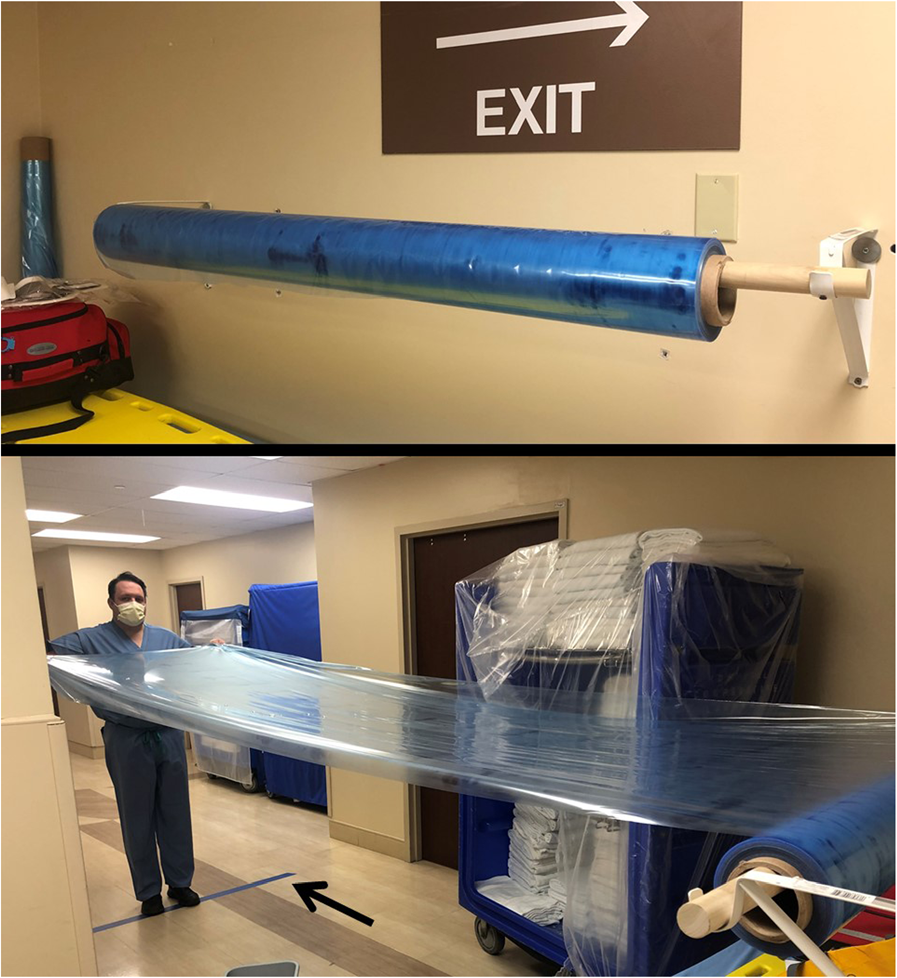
Mounting of the plastic drape roll on a designated wall in the ED (upper panel). The predetermined length of the drape is marked by a line on the floor (arrow in the lower panel)

**Fig. 5:**
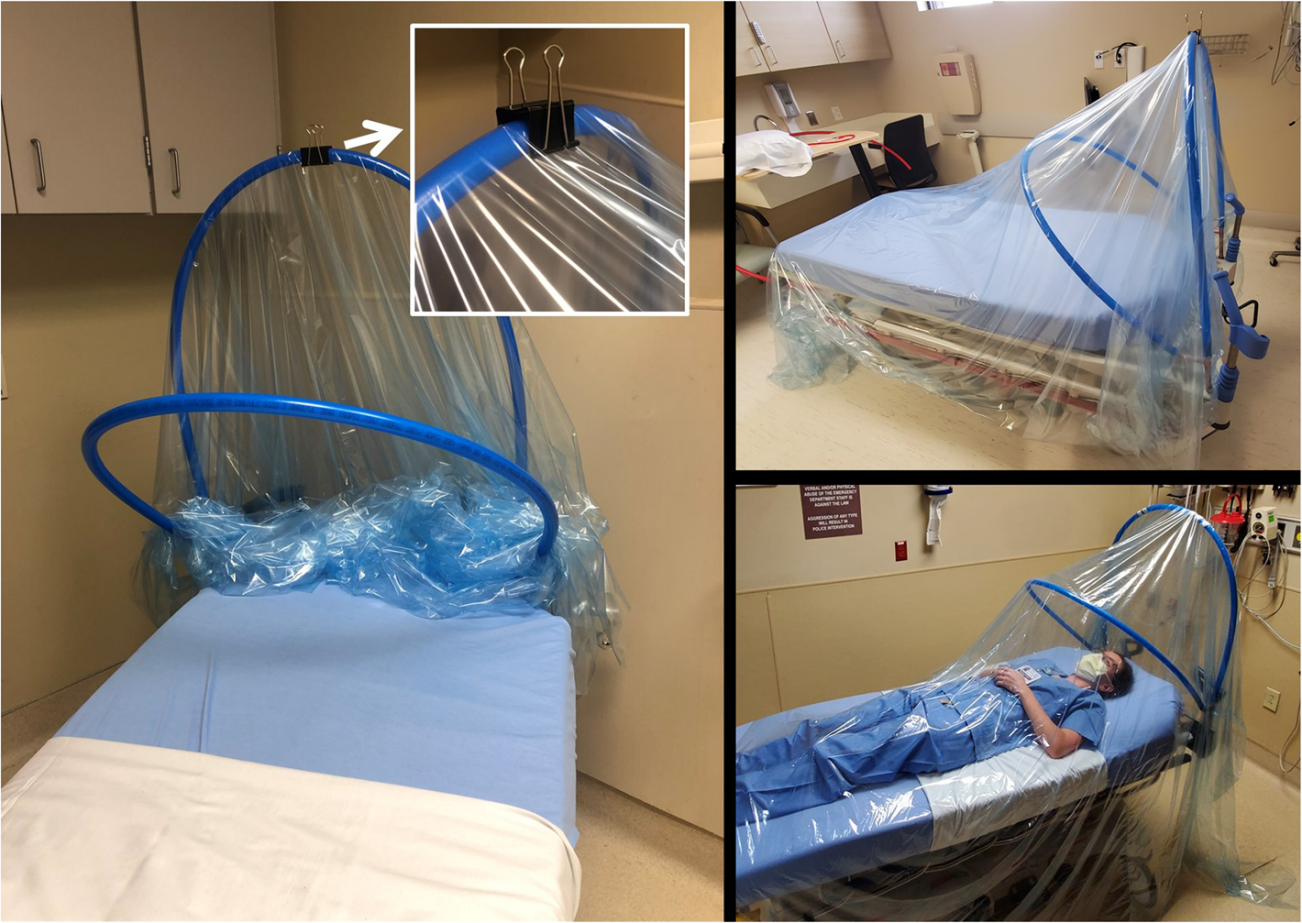
The transparent drape is attached to the proximal PEX tube with a binder clip (insert in left panel) and the pre-assembled construct is ready for use for emergent intubations in the ED bay

The copper parts are welded together for assembly of the tube post (Fig. 2, panel c). The posts are inserted to the bed through the 3/8 in. (1 cm) connecting coil tube which fits different types of commonly used patient beds in the ED (Fig. 3). The PEX tubes are then inserted to the receiving ends on the copper post, by connecting the two vertical risers and the two 45° risers each with one tube (Fig. 3). The plastic drape roll is mounted in a convenient and easily accessible place in the ED, and the predetermined length of the drape to be cut off is marked by a line on the floor (Fig. 4). The drape is attached to the proximal PEX tube with a binder clip and the construct is then ready for use in the ED bay for emergent intubations (Fig. 5).

An instructional video on how to assemble the “Corona Curtain” is available through the following link: https://youtu.be/ZQVg4b8A1NQ

### Intubation technique

The tent constructs are pre-assembled on patient beds in the ED (Fig. 5). Alternatively, the equipment can be stored in the respective ED bays for fast ad-hoc assembly within one minute. The clear plastic drapes are precut at the determined length and stored with the other tent materials. The length of the PEX tubes depends on the specific patient needs. We utilize blue PEX tubes at 6 ft length (1.8 m) for standard intubations in patients placed in supine position, which also allows to perform chest compressions, if indicated during a resuscitation. The red PEX tubes are longer, at 10 ft length (3 m), and thereby provide a larger tent size. This is helpful for situations when the patient’s head needs to be elevated, e.g. during patient transport on high-flow nasal cannula or BiPAP.

**Fig. 6:**
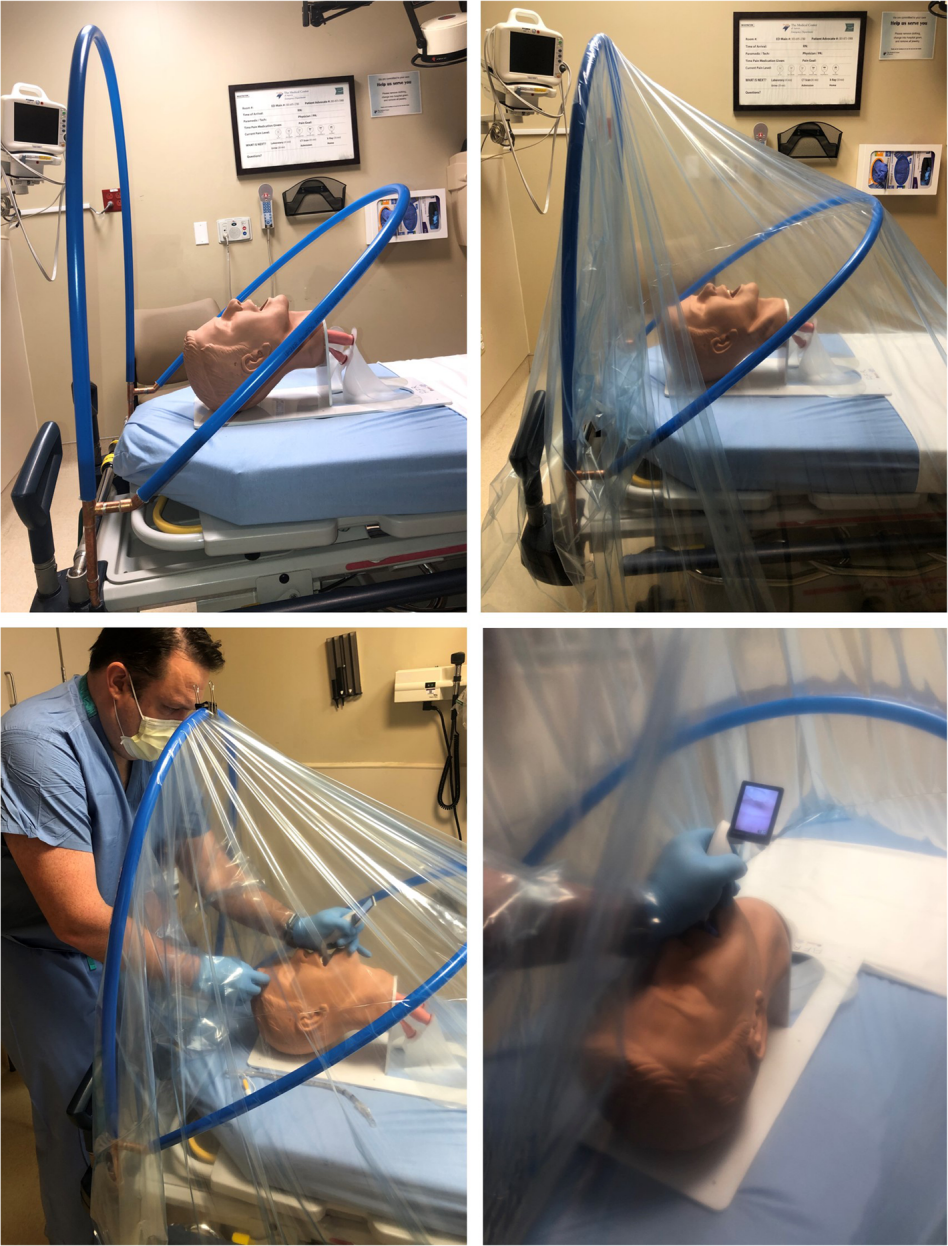
Training set-up for video-assisted laryngoscopy and intubation in a simulated airway management trainer/manikin

**Fig. 7:**
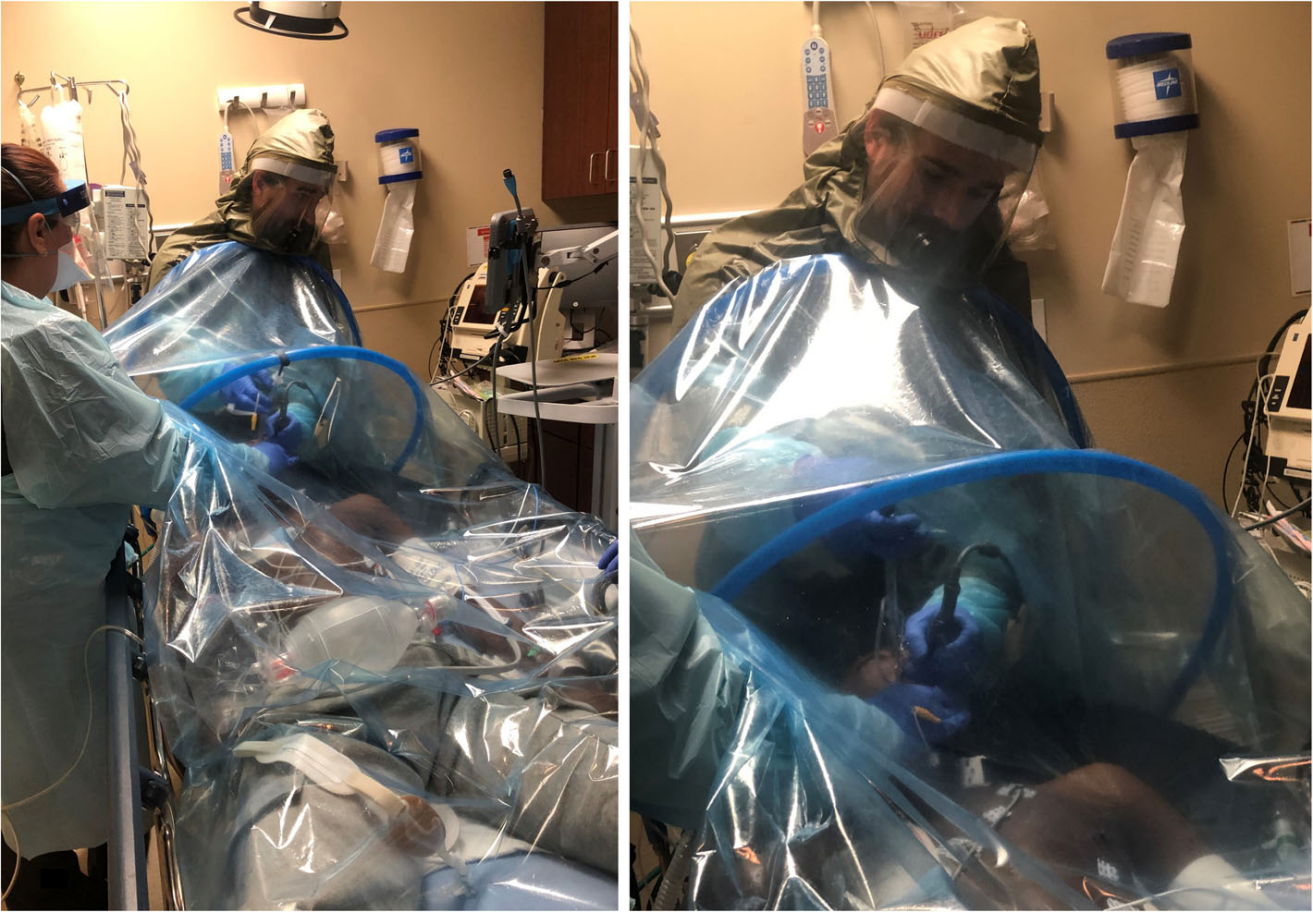
Emergent intubation using video-assisted laryngoscopy under the intubation tent in a COVID-19 patient with acute respiratory failure. The procedure is performed in a negative airflow room. The ED provider is wearing a “hazmat” suit. The respiratory therapist is assisting from the side of the bed, wearing standard PPE, N95 mask, goggles and a face shield

Once the frame is set up and the patient is positioned on the bed, the plastic drape is expanded over the PEX tubes and the patient. The edge of the drape at the head of the bed needs to extend to the floor for complete occlusion. A binder clip is applied to hold the drape to the first PEX tube which prevents the drape from sliding (Fig. 5). Subsequently, all of the work on the patient’s airway is performed under the tent (Fig. 6). Healthcare personnel can reach under the drape, and there is ample space to use a bag valve mask prior to intubating the patient, with the provider standing at the head of the bed and the nurse or respiratory therapist on the side. We recommend to utilize video laryngoscopy for improved visibility and to increase the distance between provider and patient (Fig. 6). Our ED physicians’ preference is to wear “hazmat” suits during emergent intubations (Fig. 7). The other team members wear standard PPE with N95 masks, goggles, and face shields (Fig. 7). The single-use drape is discarded after intubation and the remaining tent construct materials are terminally cleaned with bleach to be reutilized in a subsequent case.

## Preliminary experience

The novel “Corona Curtain” intubation tents were implemented in our ED at The Medical Center of Aurora, Colorado, on April 7, 2020. We assembled a total of *n* = 36 tent constructs to be deployed across the facility for emergent intubations during the COVID-19 pandemic. Our preliminary experience demonstrates that the standardized approach of using the tents in conjunction with the safety measures described above (i.e. negative airflow room; “hazmat” suit for the intubating provider; video-assisted laryngoscope; N95 masks, goggles, face shields, and PPE for the assisting nurse and respiratory therapist) works well in daily practice and has not been associated with any technical concerns or complications. From a cost perspective, the overall price of the PEX tubing, copper materials, and labor for cutting and welding of the copper posts amounted to US $ 285.00 for the first 36 tents implemented at our hospital, which extrapolates to an average price per construct of around US $ 8.00. The price of the plastic drape roll is variable depending on the specific vendor.

Our preliminary data on 25 consecutive emergent intubations for patients with acute respiratory failure or cardiac arrest in our ED between April 7 to April 25, 2020, validate the safety and feasibility of using the “Corona Curtain” in daily practice during the COVID-19 pandemic. In addition, we utilize the tent construct for patient transport when continuing aerosol-generating procedures, including high-flow nasal cannula or BiPAP, to decrease the risk of viral exposure in hallways and elevators during transport. Finally, the concept of the “Corona Curtain” may be safely extrapolated for use in the operating room and ICU during in−/extubations or other high-risk aerosol-generating procedures in SARS-CoV-2 positive patients.

## Conclusion

The “Corona Curtain” described in this article represents an intuitively pragmatic, simple, innovative and cost-effective approach to attenuating the inherent risk of aerosol exposure with potential transmission of SARS-CoV-2 to staff and providers during emergent intubations. Of note, the device has not been tested, vetted or approved by the FDA or other regulatory agencies at the time of drafting of this article. Furthermore, in spite of our positive early experience with absence of technical concerns or complications, we currently lack scientific data to define the presumed effectiveness of the “Corona Curtain”. This includes the investigational pre−/post-exposure screening for SARS-CoV-2 of patients and staff, and assessment of environmental viral contamination inside and outside of the intubation tents. Future studies will have to be designed to validate the safety and efficacy of the “Corona Curtain” during the current global COVID-19 pandemic.

## Data Availability

Please contact the authors for data requests.

## References

[1] Zhou P, Yang XL, Wang XG (2020). A pneumonia outbreak associated with a new coronavirus of probable bat origin. Nature.

[2] Rothan HA, Byrareddy SN (2020). The epidemiology and pathogenesis of coronavirus disease (COVID-19) outbreak. J Autoimmun.

[3] Heinzerling A, Stuckey MJ, Scheuer T, Xu K, Perkins KM, Resseger H, Magill S, Verani JR, Jain S, Acosta M, Epson E (2020). Transmission of COVID-19 to health care personnel during exposure to a hospitalized patient – Solano County, California, February 2020. MMWR Morb. Mortal. Wkly. Rep.

[4] Bialek S, Boundy E, Bowen V, Chow N, Cohn A, Dowling N, Ellington S, Gierke R, Hall A, MacNeil J, Patel P, Peacock G, Pilishvili T, Razzaghi H, Reed N, Ritchey M, Sauber-Schatz E, (CDC COVID-19 Response Team) (2020). Severe outcomes among patients with coronavirus disease 2019 (COVID-19) – United States, February 12-March 16, 2020. MMWR Morb. Mortal. Wkly. Rep.

[5] Burrer SL, de Perio MA, Hughes MM, Kuhar DT, Luckhaupt SE, McDaniel CJ, Porter RM, Silk B, Stuckey MJ, Walters M, (CDC COVID-19 Response Team) (2020). Characteristics of health care personnel with COVID-19 –United States, February 12-April 9, 2020. MMWR Morb. Mortal. Wkly. Rep.

[6] Guo R, Cao QD, Hong ZS, Tan YY, Chen SD, Jin HJ, Tan KS, Wang DY, Yan Y (2020). The origin, transmission and clinical therapies on coronavirus disease 2019 (COVID-19) outbreak – an update on the status. Mil Med Res.

[7] Bahl P, Doolan C, de Silva C, Chughtai AA, Bourouiba L, MacIntyre CR. Airborne or droplet precuations for health workers treating COVID-19? J. Infect. Dis. 2020; [April 16, Epub ahead of print]. 10.1093/infdis/jiaa189 PMC7184471 32301491

[8] Morawska L, Cao J (2020). Airborne transmission of SARS-CoV-2: the world should face the reality. Environ Int.

[9] Wilson NM, Norton A, Young FP, Collins DW. Airborne transmission of serve acute respiratory syndrome coronavirus-2 to healthcare workers: a narrative review. Anaesthesia. 2020; [April 20, Epub ahead of print]. 10.1111/anae.15093 PMC7264768 32311771

[10] Cook TM. Personal protective equipment during the COVID-19 pandemic – a narrative review. Anaesthesia, 2020. [April 4, Epub ahead of print]. 10.1111/anae.15158 PMC7300967 32496625

[11] Ferioli M, Cisternino C, Leo V, Pisani L, Palange P, Nava S (2020). Protecting healthcare workers from SARS-CoV-2 infection: practical indications. Eur Respir Rev.

[12] Kluge S, Janssens U, Welte T, Weber-Carstens S, Marx G, Karagiannidis C. German recommendations for critically ill patients with COVID-19. Med. Klin. Intensivmed. Notfmed. 2020; [April 14, Epub ahead of print].

[13] Wax RS, Christian MD. Practical recommendations for critical care and anesthesiology teams caring for novel coronavirus (2019-nCoV) patients. Can. J. Anaesth. 2020; [Feb 12, Epub ahead of print]. 10.1007/s12630-020-01591-x PMC7091420 32052373

[14] Rubinstein CD, DenHartog EA, Deaton AS, Bogerd CP, DeKant S (2017). Fluid replacement advice during work in fully encapsulated impermeable chemical protective suits. J Occup Envrion Hyg.

[15] Tseng JY, Lai HY. Protecting against COVID-19 aerosol infection during intubation. J Chin Med Assoc. 2020. [Epub ahead of print]. 10.1097/JCMA.0000000000000324 PMC7199771 32304507

[16] Zhang L, Li J, Zhou M, Chen Z. Summary of 20 tracheal intubations by anesthesiologists for patients with severe COVID-19 pneumonia: retrospective case series. J. Anesth. 2020; [April 17, Epub ahead of print]. 10.1007/s00540-020-02778-8 PMC7164839 32303885

[17] Kearsley R. Consensus guidelines for managing the airway in patients with COVID-19. Anaesthesia. 2020; [April 20, Epub ahead of print]. 10.1111/anae.15081 PMC7264779 32311772

[18] Cook TM, El-Boghdadly K, McGuire B, McNarry AF, Patel A, Higgs A. Consensus guidelines for managing the airway in patients with COVID-19: guidelines from the Difficult Airway Society, the Association of Anaesthetists, the Intensive Care Society, the Faculty of Intensive Care Medicine, and the Royal College of Anaesthetists. Anaesthesia. 2020; [March 27, Epub ahead of print]. 10.1111/anae.15054 PMC7383579 32221970

[19] Berkow LC, Morey TE, Urdaneta F (2018). The technology of video laryngoscopy. Anesth Analg.

[20] Stahel PF (2020). How to risk-stratify elective surgery during the COVID-19 pandemic?. Patient Saf Surg.

[21] Firstenberg MS, Libby M, Ochs M, Hanna J, Mangino JE, Forrester J (2020). Isolation protocol for a COVID-2019 patient requiring emergent surgical intervention: a case report. Patient Saf. Surg..

[22] Wen X, Li Y (2020). Anesthesia procedure of emergency operation for patients with suspected or confirmed COVID-19. Surg Infect.

[23] Rahimi F, Bezmin Abadi AT. Challenges of managing the asymptomatic carriers of SARS-CoV-2. Travel Med. Infect. Dis. 2020; [April 18, Epub ahead of print]. 10.1016/j.tmaid.2020.101677 PMC7165291 32315756

[24] Zareifopoulos N, Lagadinou M, Karela A, Karantzogiannis G, Velissaris D (2020). Intubation and mechanical ventilation of patients with COVID-19: what should we tell them?. Monaldi Arch Chest Dis.

[25] Giwa AL, Desai A, Duca A (2020). Novel 2019 coronavirus SARS-CoV-2 (COVID-19): an updated overview for emergency clinicians. Emerg Med Pract.

